# ‘Hitting a brick wall’—the importance of careful pacemaker programming

**DOI:** 10.1093/omcr/omaf272

**Published:** 2025-12-26

**Authors:** Ankit Gupta, Charlotte Cole, Sam Straw, Stephe Kamalathasan, John Gierula

**Affiliations:** Leeds Teaching Hospitals NHS Trust, Great George Street, Leeds, West Yorkshire, LS1 3EX, United Kingdom; Leeds Teaching Hospitals NHS Trust, Great George Street, Leeds, West Yorkshire, LS1 3EX, United Kingdom; Leeds Institute of Cardiovascular and Metabolic Medicine, University of Leeds, Woodhouse Lane, Leeds, West Yorkshire, LS2 9JT, United Kingdom; Leeds Institute of Cardiovascular and Metabolic Medicine, University of Leeds, Woodhouse Lane, Leeds, West Yorkshire, LS2 9JT, United Kingdom; Leeds Institute of Cardiovascular and Metabolic Medicine, University of Leeds, Woodhouse Lane, Leeds, West Yorkshire, LS2 9JT, United Kingdom

**Keywords:** cardiac resynchronisation, electrocardiogram, pacemaker

## Abstract

**Background:**

Pacemakers attempt to accurately mirror normal physiology; however, even normal pacemaker functionality can have undesirable effects.

**Case Summary:**

A 59-year-old male with a background including heart failure, presented with symptoms of ‘hitting a brick wall’ during exertion, years following the implantation of a biventricular pacemaker for third-degree atrioventricular block. He was extensively evaluated, with cardiopulmonary exercise testing and simultaneous device interrogation. After reaching a heart rate of 140 beats/minute, continued exercise resulted in Wenckebach phenomenon and progression to sustained 2:1 atrioventricular block with immediate reductions in oxygen consumption. Ultimately, the aetiology was attributed to upper rate behaviour, rather than the progression of heart failure, and resolved with simple device reprogramming.

**Discussion:**

Cardiopulmonary exercise testing is an invaluable tool when evaluating patients who present with cardiovascular symptoms. Device reprogramming is often required, including the use of rate-response algorithms where appropriate. Personalisation is key, with programming tailored to the individual’s physiology.

## Introduction

We report the case of a 59-year-old male patient who underwent evaluation for atypical symptoms during physical exertion. We explored the functionality of a cardiac resynchronisation therapy-pacemaker (CRT-P) device during cardiopulmonary exercise testing (CPEX). This case is distinct due to several reasons: the patient’s complex medical background, the long interval between pacemaker implantation and the onset of symptoms, and the integration of CPEX with device interrogation to determine the cause of symptoms. Furthermore, this case highlights how careful device programming, aligned to an individual’s physiology can result in dramatic functional improvements [1].

## Case report

A 59-year-old male presented with symptoms of exercise intolerance and breathlessness on exertion. The timeline of events is summarised in Table 1. Past medical history included Wolff-Parkinson-White syndrome which, at 21-years-old, resulted in out-of-hospital cardiac arrest due to ventricular fibrillation. This event pre-dated the widespread availability of transcatheter ablation techniques, and so was managed by open surgical division of the accessory pathway. The operation was complicated by the development of third-degree atrioventricular block, for which a dual-chamber pacemaker was implanted.

**Table 1:** Timeline of clinical events.

Age (years)	Event
21	Out-of-hospital ventricular fibrillation cardiac arrest due to Wolff-Parkinson-White syndrome.
	Open surgical division of the accessory pathway.
	Dual-chamber pacemaker implanted due to operative complication of third-degree heart block.
53	Device upgraded to CRT-P due to development of LVSD and HF symptoms.
	LVEF approximately 35%.
59	Development of exercise intolerance symptoms—‘hitting a brick wall’ during exertion.
	Device re-programming.
	Initiated on sacubitril-valsartan 97/103 mg and bisoprolol 1.25 mg.
	Follow-up LVEF 45–50%.

Key: CRT-P = cardiac resynchronisation therapy pacemaker); LVSD = left ventricular systolic dysfunction; HF = heart failure; LVEF = left ventricular ejection fraction.

At 53-years-old, the patient developed pacing-associated left ventricular systolic dysfunction (LVSD) and symptoms of heart failure (HF); a decision was made to upgrade to a CRT-P device (Medtronic Viva CRT-P Model C5TR01). The patient’s symptoms subsequently resolved, and he remained well despite not receiving guideline-directed medical therapy (GDMT) for HF due to several drug intolerances.

Five years later, the patient re-presented to our institution due to ‘hitting a brick wall’ during strenuous exercise. The patient reported a predictable pattern of breathlessness and fatigue, especially whilst exercising on a treadmill.

A transthoracic echocardiogram showed a LV ejection fraction (LVEF) of approximately 35%. Although initial investigations supported a presumptive diagnosis of progressive LV remodelling, as well as symptoms which might be attributed to pharmacologically untreated HF, in view of the atypical description, we comprehensively evaluated the patient with CPEX and simultaneous interrogation of device programming and functionality.

The patient’s device was programmed to a dual-chamber pacing mode with a base rate of 40 beats/minute (DDD-40). Heart rate histograms showed no evidence of chronotropic incompetence; there were no alerts for atrial or ventricular high-rate episodes, and no mode switching episodes recorded by the device.

Exercise was undertaken using a ramping treadmill protocol with a maximum speed of 4 km/hour and a maximum gradient of 6%, with simultaneous 12-lead electrocardiography. The patient remained asymptomatic until approximately nine minutes, at which point he reported the sudden onset of symptoms, and exercise was terminated at his request.

**Figure 1:**
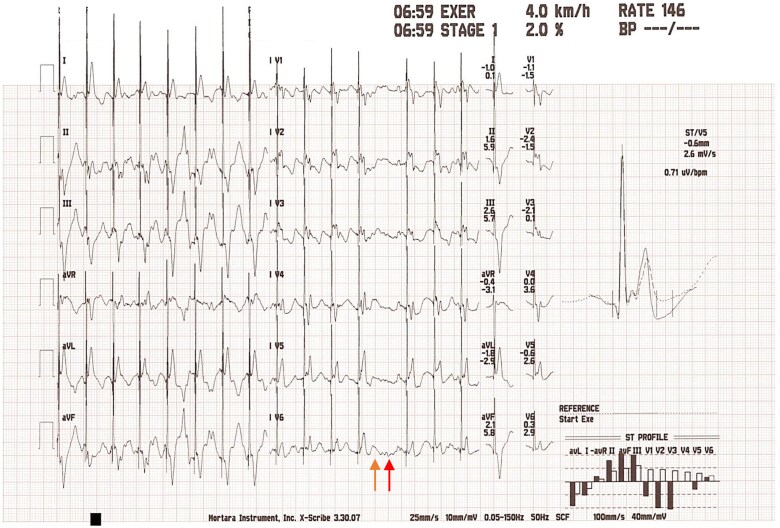
ECG at five minutes of exercise. The first appearances of Wenckebach phenomena are demonstrated, whereby a QRS complex is omitted (red arrow) following a P wave (orange arrow).

**Figure 2:**
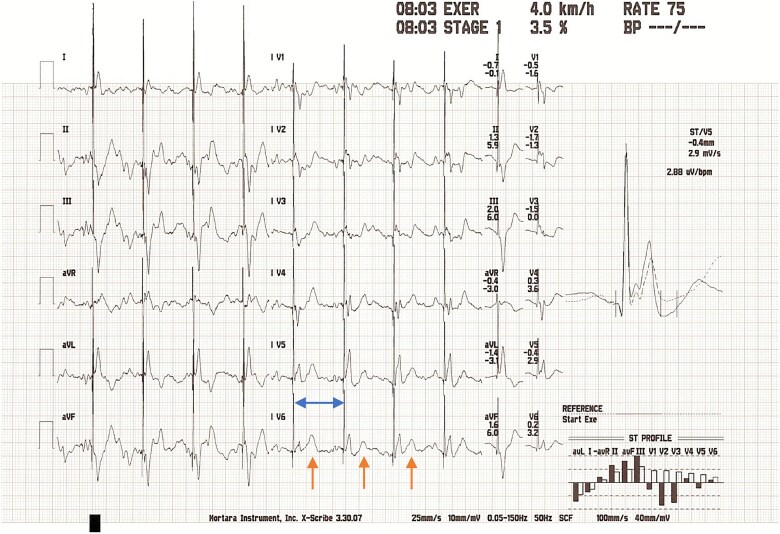
ECG at six minutes of exercise. Appearances are consistent with a sustained 2:1 block, whereby every alternate P wave (orange arrow) is not followed by a QRS complex. The R-R interval is 600 milliseconds (blue arrow).

The baseline electrocardiogram demonstrated an atrial sensed, biventricular paced rhythm at 66 beats/minute. During exercise, the patient’s sensed atrial rate increased to 140 beats/minute and the electrocardiogram continued to demonstrate an atrial sensed, biventricular paced rhythm. Beyond this, incremental exercise resulted in progressive prolongation of the PR interval until a P wave was not followed by a biventricular paced QRS complex (Fig. 1). This then progressed into a sustained 2:1 atrioventricular block (Fig. 2), with a reduction in the paced ventricular rate to approximately 75 beats/minute. During recovery, the paced ventricular rate increased as the intrinsic atrial rate reduced, before returning to resting values.

**Figure 3:**
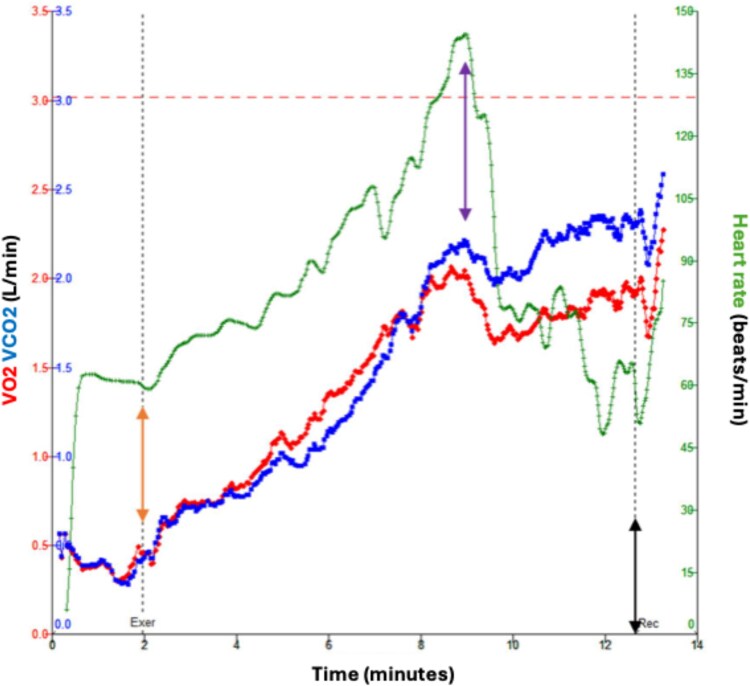
VO_2_, VCO_2_, and heart rate plotted against time (minutes). Exercise was commenced at 2 minutes, where a physiological increase in heart rate, VO2, and VCO2 rise were observed (orange arrow). Wenckebach phenomena was observed around 9 minutes of exercise, following which a decrease in heart rate, VO2, and VCO2 were seen (purple arrow). Exercise was ceased just after 12 minutes due to patient fatigue (black arrow).

The development of atrioventricular block was also associated with a reduction in oxygen consumption (Fig. 3). Post-maximum pacing rate, the V̇O2 and V̇CO2 reduced, suggesting a decline in metabolic activity and oxygen uptake efficiency due to the sudden reduction in cardiac output.

The cause of our patient’s symptoms was ultimately attributed to upper rate behaviour. The upper tracking rate was reprogrammed from 140 to 170 beats/minute. Biventricular pacing was 99.2%. Attempts were made to modify V-V delays, however this did not result in any improvement in the QRS duration. The patient was then initiated on sacubitril-valsartan 97/103 mg and bisoprolol 1.25 mg, although declined further intensification of GDMT. At follow-up he has a LVEF of 45–50% and reports New York Heart Association classification I symptoms.

## Discussion

We report the case of a 59-year-old male who presented with exercise intolerance, which might otherwise have been attributed to the syndrome of HF in the context of pacing-associated LVSD and the absence of GDMT. However, detailed evaluation with CPEX and device interrogation revealed symptoms attributable to upper rate behaviour, which weremanaged by simple device reprogramming.

Personalised pacemaker reprogramming is critical in managing symptoms related to upper rate behaviour. A previous study found that personalised reprogramming of pacemakers in patients with bradycardia can result in improvements in cardiac function, and extend battery longevity, with no detriment to quality of life [1]. Right ventricular pacing is associated with an increased risk of HF, however this could not be avoided in our patient due to complete heart block [2]. The 2021 European Society of Cardiology guidelines advocate that pacemaker programming should be tailored to meet patients’ needs [3]. Recent studies also highlight the role of remote monitoring and iterative reprogramming, allowing for longitudinal refinement of pacing parameters to accommodate evolving patient physiology and comorbidities [4–6].

Although our patient experienced significant symptomatic improvement following CRT-P reprogramming, it is important to emphasise that GDMT remains the foundation of HF management. GDMT improves survival, reduces hospitalisations, and promotes reverse remodelling [7]. In our case, drug intolerance limited escalation of therapy, and the patient declined further intensification. Device optimisation therefore played a pivotal role in alleviating symptoms, evidenced by their rapid resolution with simple reprogramming. In all cases device optimisation should be regarded as complementary rather than substitutive to pharmacological therapy.

CPEX is an indispensable tool in the evaluation of patients with HF, especially when new cardiorespiratory symptoms arise which cannot be attributed to another common pathology. In our case, transthoracic echocardiography excluded overt structural pathology and device interrogation excluded arrhythmia. CPEX was chosen because it uniquely integrates cardiovascular, respiratory, and metabolic responses, allowing precise correlation of exercise limitation with device behaviour, with metrics such as peak V̇O2 serving as robust predictors of clinical outcomes [8]. Furthermore, the ventilatory equivalent for carbon dioxide (V̇E/V̇CO2 slope) derived from CPEX has been demonstrated to be a strong independent predictor of mortality in patients with HF, with higher slopes indicating poorer prognosis (9). Alternative modalities such as stress echocardiography lack such physiological granularity, whilst invasive haemodynamics are more cumbersome and come with associated risks.

The CPEX data indicate that our patient’s anaerobic threshold occurred around 110 beats/minute, preceding the onset of atrioventricular block. With the prior upper tracking rate of 140 beats/minute, the patient was limited in achieving heart rates above this threshold during exertion. Increasing the upper tracking rate to 170 beats/minute allowed the device to support higher heart rates during exercise. However, programming a higher upper tracking rate in a patient with HF, broad QRS, prior cardiac arrest, history of heart surgery, and suboptimal GDMT carries safety considerations, including the risk of precipitating arrhythmias or worsening heart failure. In this context, CPEX can help guide a tailored approach by identifying the heart rate range associated with effective exercise without inducing ischaemia or hemodynamic compromise. Additionally, CPEX findings can inform personalised physical activity recommendations, allowing safe exercise prescriptions and monitoring of functional improvement. While a follow-up CPEX was not performed for our patient, it may be considered in future assessments to evaluate the effect of device optimisation on exercise capacity and chronotropic response.

## Conclusions

This case highlights the role of CPEX in symptom evaluation and the importance of personalised pacemaker programming. By extensively evaluating our patient, we demonstrate how CPEX can identify specific cardiac responses and limitations imposed by pacemaker upper rate settings. Personalised programming may be essential to address unique physiological demands.
